# Replanting Teeth

**Published:** 1903-05-15

**Authors:** G. S. Hadley


### REPLANTING TEETH.

BY G. S, HADLEY, D.D.S.

Read before the Southwestern Michigan Dental Society, April 8, 1903.

My first experience in replantation was in the spring of 1898. A working man whose tooth had never seen a toothbrush came to my office. His gums were swollen, tobacco-stained and inflamed. He was suffering great pain with a left superior lateral. It was so sore that the slightest touch caused him excruciating pain. After vainly trying to explore a cavity in the tooth, I finally extracted it and to my amazement found a piece of a tooth-pick extending through the apical foramen. I then suggested to him the idea of replacing it. I had never undertaken anything of the kind before and told him it was simply a matter of experiment, but if he wanted to save the tooth we would try it, and the method that I followed then I have followed with very little variation since.

I thoroughly sterilized the tooth itself with carbolic acid, oils of clove and cinnamon, also being careful to sterilize my hand while handling the tooth, cleaned out the cavity and filled the root canal. After thoroughly washing the tooth socket with hydrogen dioxide, I then placed a pledget of cotton, soaked in strong cocain in the tooth socket and let it remain until it became numb. I then cleansed out the socket with oil of cloves and replaced the tooth.

In preparing the tooth I ground off a little at the end of the root, scraping the loose membranes from the root, being careful not to injure the peridental membranes, After replacing the tooth I ligatured it to the central and cuspid. I had the patient cleanse the mouth three times daily with listerine, left the ligature in place for ten days, and on removing it at the end of that time, I found the tooth perfectly solid.

But a few months ago I met the gentleman and on asking him how the tooth was, he had to stop and feel of first one tooth and then another before he found out which tooth it was, and it was apparently as solid as any tooth in his head.

Another case which came to me, was that of a young lady. The whole of the gum tissue surrounding a lateral incisor was infiltrated with pus, and apparently the abscess had destroyed all of the alveolar process. The tooth could almost be removed with the fingers. The patient wanted me to remove it and put in a bridge. A year and a half or two years before she had been to a dentist and the tooth was abscessed and he made an opening through the tooth and then injected hydrogen dixoide into this pus cavity through the tooth. You can readily imagine the consequence. The patient said she believed she had hysterics and I have no doubt she did. For a year and a half she let that tooth go rather than go to a dentist again. Her sufferings had been so terrible. The hydrogen dioxide had driven the pus into the surrounding membranes until there were three or four openings on to the gum, where the slightest pressure caused the pus to flow.

I carefully removed the tooth, scraped the root in the rough places where the abscess had destroyed the membranes thoroughly cleansed the tooth socket cutting away considerable of the cicitricial tissue and cleansing it several times with hydrogen dioxide. I then thoroughly cleansed it with oil of cloves, replaced the tooth and ligatured it to the central and cuspid. I allowed these ligatures to remain for two weeks and then replaced them with new ligatures and allowed them to remain for two weeks more, having the patient thoroughly cleanse the mouth three times daily with listerine. I know a new bone tissue was formed here. The tooth is solid to this day. The patient uses it and does not notice any difference from the other teeth.

By this same method I have replanted superior and inferior molars and superior and inferior bicuspids and incisors. Possibly, the roots may absorb in time, but at present none of those that I have replanted, three and four years ago, have shown the least signs of loosening.

Last November, a young boy had a tooth knocked out in a game of foot ball. The next morning he brought it to the office in his pocket. After some persuasion I induced him to allow me to replant it. The tooth is not as solid to-day as the other ones, but it still does him good service, and I believe will, in time, become perfectly solid.

While in the general practice there may not be a very large field for replanting teeth, still in a great many instances I am sure that teeth are lost whose life might be prolonged a great many years by replanting. It certainly can be successfully done if we are careful to perfectly sterilize everything.

I believe that oil of cloves or Black's "one-two-three" remedy is as good for this as anything that can be obtained. While sterilizing the teeth, it does not cauterize.

There certainly must be some life in a tooth after extraction, which becomes active again after the tooth is replanted.

I have known in one instance of the burr being used on the teeth and the patient claims to have felt it.

And if the old saying is true, that the loss of a tooth shortens a person's life a year, then if we save a tooth it should prolong a life at least one year.

### DISCUSSION.

Dr. N. E. Hooper: My experience in replantingteeth is not extensive. Twenty-seven years ago a boy came to me with a central incisor broken off below the gum. I removed the root and at the suggestion of my patient, attached a porcelain crown with a wooden peg to the root and replanted it. There was in due time a firm union and the tooth gave good service for years. The crown finally became loose and the patient, being a carpenter, reset it himself several times. Ten years ago he came to me with the crown so loose that he could not make it stay with his wood pegs. I reset it with amalgam around the pin. The tooth is still in place and doing good service.

Some years ago a lady came to me with an abscessed lateral incisor, the pulp canal having been previously filled with amalgam. In removing the amalgam I perforated the root canal on the side. This caused a fleshy tumor to develop on the side of the root which would not submit to treatment. I extracted the tooth, cut off the end of the root and filled the canal with gold and replanted the tooth. The tooth became firmly attached and did good service for ten years. I used only carbolized water to sterilize the field of operation and the root.

I have made several other attempts at replanting, but have not had satisfactory results.

Dr. W. D. Campbell: A gentleman came to my office recently with a tooth that had been replanted by a dental student. It was a crude operation and probably little or no care was taken to observe antiseptic precautions. Still the tooth seemed to be firmly attached and had been in place about two years.

Dr. E, M. Cook, of Toledo: I had a tooth replanted in my own mouth by Dr. Younger, which remained in place but six weeks and I suffered almost intolerable pain during the whole time.

I have had a number of successful cases of replanting in my practice, also a goodly number of complete failures. I have one case that is standing over twelve years. I know of a case where a street-car man got hit in the mouth with a trolley pole and one central incisor was broken off, while the other was knocked completely out and fell down on to the paved street into the dirt. He climbed down from the roof of the car and found his tooth and washed it in his own tobacco spittle and pushed it back into the socket, it became firmly attached and is doing good service.

DR. C. B. ROE: I have done some replanting, and have had several cases which I count as successful although five years is the longest time any of my cases have lasted. In most of these cases the roots absorb in two or three years. But if we can prolong a tooths existence three or five years by replanting, I am inclined to call it a successful practice.

DR. J. H. PALIN: Dr. Hutchinson, a U. of M. graduate of 1884, who is now practicing in Honolulu, told me that he did a great deal of replanting, that he would average from eight to ten replantations each week He claimed to have general success in the operation. He thought possibly that climatic conditions on the island favored the operation, but he could not tell in what way.

DR. C. B. CHADWICK: While a student in college I had accidentally extracted a lower second bicuspid in attempting to extract the molar just back of it. I immediately replanted the bicuspid and it is still in place and doing good service. I have made many operations since, but have not had sufficient success to warrant me in being very enthusiastic about this operation. I think I shall try the tobacco-juice antiseptic some time.

DR. GEO. COOK, of Chicago: A student in the Illinois Dental College in extracting some teeth for a patient under the influence of nitrous oxide gas, extracted a bicuspid by mistake and threw it into the cuspidore. When he discovered his mistake he recovered the tooth and replanted it without any sterlizing or other treatment. He kept track of the patient and the tooth is still in place and doing good service after two years.

Implantation, replantation and transplantation have all been done with some degree of success, but probably a larger percentage of replantation is successful than either of the other operations.

I have replanted between twenty-five and thirty teeth, and I should say that probably fifty percent of the cases have remained in good condition for from five to six years.

I am not able to say what conditions are most favorable to success. I am of the opinion that plastic fillings, which most perfectly seal the tooth, are most likely to be successful. A confrere of mine tells me that of seven teeth which he implanted, those which had gold root fillings seem to have absorbed more quickly than those filled with gutta-percha.

At a clinic before the Illinois Dental Society, twelve years ago, a tooth was extracted for a young lady fifteen years of age to illustrate the anesthetic value of cocain. A dentist present thought it a shame to lose so good a tooth, so he filled it and replanted it, using no special antiseptic precautions. I have watched that tooth since then and it is still doing good service and shows no signs of degeneration.

There is on record in old literature a statement that the old Romans used to have their decayed teeth replaced by the good ones taken from the mouths of their slaves. Dr. Younger, as is well known, implants teeth, using teeth that have been extracted for months or even years and claims to have good results.

A young lady patient of mine by an accident fractured a lateral incisor beyond the possibility of repair. I secured a tooth to match the space as nearly as possible and implanted it. It is a success so far as the attachment is concerned. The color is not as good as I should like.

I think it a good plan to tabulate our cases and keep accurate records so that we may eventually have sufficient statistics to enable us to come to some conclusion as to the value of such operations.

I find that teeth which have been badly abscessed or necrosed are not likely to produce successful results.

Dr. Hadley, in closing, related a case which came under his observation of a tooth which had been knocked out of the jaw by the kick of a horse. The patient, a young man, picked the tooth up out of the sand and replaced it in its socket. It became firmly attached without any treatment and gives every indication of being a live tooth. Dr. Welch tells me that he notches the roots so that by a deposit of bone the tooth is held more securely in the jaw. This I have never done.

